# Humanized MISTRG as a preclinical *in vivo* model to study human neutrophil-mediated immune processes

**DOI:** 10.3389/fimmu.2023.1105103

**Published:** 2023-03-08

**Authors:** Paula Martinez-Sanz, Adrien R. G. Laurent, Edith Slot, Mark Hoogenboezem, Nikolina Bąbała, Robin van Bruggen, Anthony Rongvaux, Richard A. Flavell, Godelieve A. M. Tytgat, Katka Franke, Hanke L. Matlung, Taco W. Kuijpers, Derk Amsen, Julien J. Karrich

**Affiliations:** ^1^ Sanquin Research and Landsteiner Laboratory, Department of Molecular Hematology, Amsterdam UMC, University of Amsterdam, Amsterdam, Netherlands; ^2^ Sanquin Research and Landsteiner Laboratory, Department of Hematopoiesis, Amsterdam UMC, University of Amsterdam, Amsterdam, Netherlands; ^3^ Department of Immunology, University of Washington, Seattle, WA, United States; ^4^ Fred Hutchinson Cancer Research Center, Clinical Research Division, Seattle, WA, United States; ^5^ Department of Immunobiology, Yale University School of Medicine, New Haven, CT, United States; ^6^ Howard Hughes Medical Institute, Yale University School of Medicine, New Haven, CT, United States; ^7^ Princess Maxima Center for Pediatric Oncology, Department of Pediatric Oncology, Utrecht, Netherlands; ^8^ Rheumatology and Infectious Diseases, Emma Children's Hospital, Department of Pediatric Immunology, Amsterdam UMC, University of Amsterdam, Amsterdam, Netherlands

**Keywords:** neutrophils, animal model, humanized immune system mouse, next generation humanized mouse models, preclinical study, MISTRG

## Abstract

**Introduction:**

MISTRG mice have been genetically modified to allow development of a human myeloid compartment from engrafted human CD34+ haemopoietic stem cells, making them particularly suited to study the human innate immune system *in vivo*. Here, we characterized the human neutrophil population in these mice to establish a model that can be used to study the biology and contribution in immune processes of these cells *in vivo*.

**Methods and results:**

We could isolate human bone marrow neutrophils from humanized MISTRG mice and confirmed that all neutrophil maturation stages from promyelocytes (CD11b–CD16–) to end-stage segmented cells (CD11b+CD16+) were present. We documented that these cells possessed normal functional properties, including degranulation, reactive oxygen species production, adhesion, and antibody-dependent cellular cytotoxicity towards antibody-opsonized tumor cells *ex vivo*. The acquisition of functional capacities positively correlated with the maturation state of the cell. We found that human neutrophils were retained in the bone marrow of humanized MISTRG mice during steady state. However, the mature segmented CD11b+CD16+ human neutrophils were released from the bone marrow in response to two well-established neutrophil-mobilizing agents (i.e., G-CSF and/or CXCR4 antagonist Plerixafor). Moreover, the neutrophil population in the humanized MISTRG mice actively reacted to thioglycolate-induced peritonitis and could infiltrate implanted human tumors, as shown by flow cytometry and fluorescent microscopy.

**Discussion:**

These results show that functional human neutrophils are generated and can be studied *in vivo* using the humanized MISTRG mice, providing a model to study the various functions of neutrophils in inflammation and in tumors.

## Introduction

Pre-clinical mouse models are essential for the understanding of human physiology, and immunity. There are, however, many differences between mice and humans (1), and findings derived from laboratory animals cannot always be directly translated to humans (2, 3). The use of immunodeficient mice grafted with human hematopoietic stem cells (generally referred to as ‘humanized mice’) is, among others, a promising approach for studying human immune development and function *in vivo* (2, 4, 5). Traditional humanized mouse models have been unable to reliably establish a human myeloid compartment, limiting study of especially innate human immune functions (2, 6, 7). Immunocompromised *NOD/scid/IL2Rγ^–/–^* (NSG) mice exhibit largely defective development of human monocytes/macrophages and NK cells after human immune reconstitution with CD34^+^ hematopoietic progenitor cells (HPC) (6, 8, 9), likely due to the limited cross-reactivity between specific mouse and human cytokine receptors (2, 7).

The delivery of human cytokines to humanized mice *via* the knock in of cytokine-encoding genes can circumvent this challenge (10–13). Building on the highly immunodeficient *Rag2^–/–^/IL2Rγ^–/–^* background, Rongvaux et al. generated an improved model in which the human genes encoding macrophage colony-stimulating factor (M-CSF), interleukin-3 (IL-3), granulocyte-macrophage colony-stimulating factor (GM-CSF) and thrombopoietin (TPO) were knocked in to replace their mouse counterparts (6). These cytokines were strategically chosen: they have critical roles in early hemato- and myelopoiesis, but the human receptors for these cytokines do not respond to the corresponding murine cytokines. An elegant consequence of the improved early human hematopoiesis is that the generated human cell types produce additional human cytokines (such as IL-15), which further bolsters human hematopoiesis (6). Moreover, a Bac transgene was introduced to express the human signal-regulatory protein alpha (SIRPα), which promotes acceptance of human xenografts in mice by inhibiting phagocytosis by mouse macrophages (14). This mouse strain was named ‘MISTRG’, and was shown to allow successful development of not only lymphoid T and B cell compartments, but also of diverse and functional human myeloid and NK cells, resembling those seen in human blood. Human NK cells from humanized MISTRG (huMISTRG) mice were fully functional and exhibited cytotoxic activity towards human tumor cells (6, 15), and human macrophages were shown to infiltrate human tumor xenografts in a pattern resembling that observed in tumors from human patients (6). huMISTRG mice also supported the development of human neutrophils, among other granulocytes (6, 16).

Neutrophilic granulocytes play pivotal roles in host immune defense. They constitute an important early barrier to invasion by infectious agents through mechanisms including phagocytosis, the release of reactive oxygen species (ROS), antimicrobial peptides and proteases, as well as through antibody-mediated mechanisms such as antibody-dependent cellular cytotoxicity (ADCC) (17–19), among others. In addition, recent evidence has made clear that neutrophils can also act as danger sensors and, by interacting with other immune cells, contribute to the establishment of adaptive immune responses (20). Hence, dysregulation of neutrophils can lead to a variety of pathologies (21). Neutrophils can both have beneficial as well as adverse functions. An example of this is in cancer, where neutrophils eliminate cancer cells, but also oppose immune control of tumors by differentiating into myeloid-derived suppressor cells (18, 19). It is therefore important to establish pre-clinical models where the biology of human neutrophils and their involvement in health and disease can be studied.

Despite being present in the bone marrow of huMISTRG mice, the frequency of neutrophils in peripheral blood of such mice is negligible. It has been suggested that either the terminal differentiation, the egress from the bone marrow or the peripheral survival of human neutrophils are still suboptimal in this mouse environment (6). Still, a subset of human neutrophils in huMISTRG mice was found to possess a mature phenotype as described by a CD33^+^CD66b^+^CD16^+^ surface phenotype and the presence of segmented nuclei (16). Here, we aimed to further characterize the human neutrophil population to validate huMISTRG mice as an experimental model to study neutrophil biology and their contribution in various immune processes *in vivo*. We successfully isolated both immature and end-stage human neutrophils from huMISTRG mice and assessed their functionality in a number of neutrophil-specific assays. Moreover, we showed that they could be mobilized into blood in response to two well-established neutrophil-mobilizing agents (*i.e.*, granulocyte colony-stimulating factor and/or CXCR4 antagonist Plerixafor), and were actively recruited to inflammation sites induced by thioglycolate as well as by human tumors engrafted *in vivo*.

## Materials and methods

### Human immune reconstitution of immunodeficient mice

Highly immunodeficient MITRG (M-CSF^h/h^ IL-3/GM-CSF^h/h^ TPO^h/h^ Rag2^–/–^ IL2Rγ^–/–^) and MISTRG (M-CSF^h/h^ IL-3/GM-CSF^h/h^ hSIRPAtg TPO^h/h^ Rag2^–/–^ IL2Rγ^–/–^) mice were generated as described before (Regeneron (6), and maintained under specific pathogen free conditions with continuous Enrofloxacin antibiotic treatment in drinking water (Baytril, 0,27 mg/mL; Bayer).

**Figure 1:**
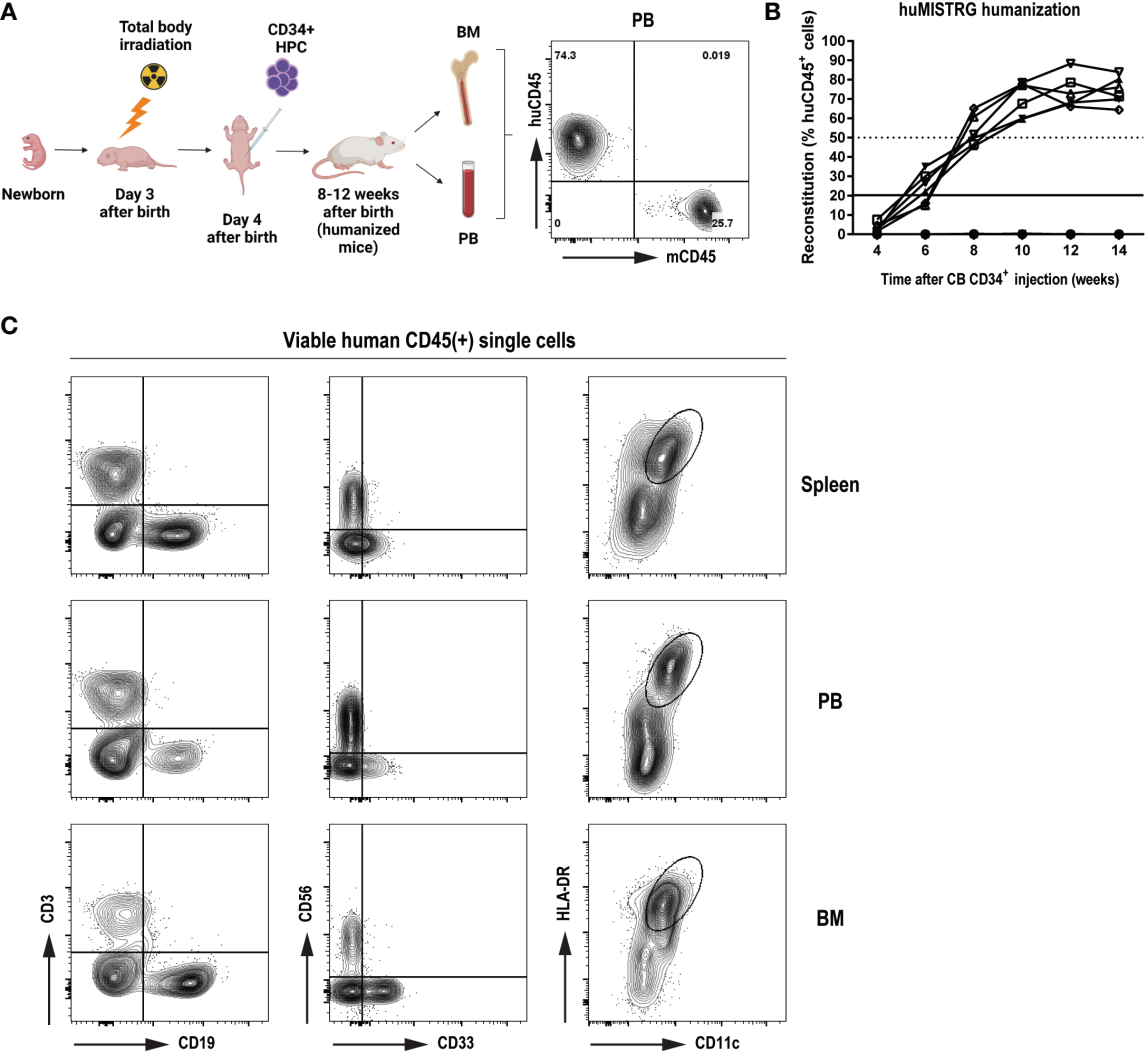
General characterization of human immune compartment in huMISTRG mice **(A)** Schematic representation of human reconstitution procedure in huMISTRG mice until harvest of the material. Animals undergo sublethal total body irradiation on day 3 after birth, one day prior to intraperitoneal injection of CD34^+^ HPC. Adult animals of 8-12 weeks of age are checked for successful humanization (huCD45 vs mouse CD45) and organs (*i.e.*, BM, PB) are harvested for subsequent experiments. Numbers indicate the percentages of the gated populations. Created with BioRender.com. **(B)** Kinetics of human reconstitution as determined by % of human CD45 cells after cord blood CD34^+^ cell injection of 10 independent mice. **(C)** Representative flow cytometry analysis of human immune characterization (CD3^+^ T lymphocytes, CD19^+^ B lymphocytes, CD56^+^ NK cells, CD33^+^ myeloid cells, and CD11c^+^HLA-DR^+^ dendritic cells) gated on viable human CD45^+^ single cells 8 weeks after transplantation in spleen (top), PB (middle) and BM (bottom) compartments of huMISTRG mice. HPC, hematopoietic progenitor cells; CB, cord blood; PB, peripheral blood; BM, bone marrow.

Newborn MISTRG mice (within first 3 days after birth) were sublethally irradiated (X-ray irradiation with Faxitron MultiRad 225, 10 cGy), and were subsequently injected intrahepatically with 1 × 10^5^ cord blood (CB)-derived CD34^+^ cells (CB was collected according to the guidelines of Eurocord Nederland), unless otherwise specified. Level of human immune reconstitution was measured from week 4 after CD34^+^ cell engraftment using flow cytometry on blood samples (percentage of human CD45^+^ cells, as compared to percentage of murine CD45^+^ cells within total CD45 immune cells; BV421-labeled anti-human CD45 (clone HI30; BioLegend), PE-Cy7-labeled anti-mouse CD45 (clone 30-F11; BioLegend) (**Figure 1A**). Mice with at least 20% huCD45^+^ cells in the blood were selected for further experiments. Of note, each experimental replicate presented in this study was performed with a cohort of huMISTRG generated with a different human CB donor.

### Tissue sampling

Prior to tissue sampling, mice were sacrificed by carbon dioxide asphyxiation. Peripheral blood samples were harvested by heart puncture with syringe and needle. Contaminating erythrocytes were removed using red blood cell lysis buffer (155 mM NH4Cl, 10 mM KHCO3, 0.1 mM EDTA). For the harvesting of bone marrow cell suspension, femur and tibia from both legs were harvested and bones were crushed in PBS supplemented with 0.5% (v/v) fetal calf serum (FCS) with a pestle and mortar. For the removal of fibrous tissue, cell suspension was passed through a 100 μm mesh. Single cell suspensions from spleen were prepared by mechanical disruption *via* passing of the tissues over a 70 μm cell strainer. Tumor tissue was cut into pieces of 1 mm2 and enzymatically digested for 30 min at 37°C with 750 U ml-1 Collagenase Type I (Worthington) and 0.31 mg ml-1 DNase I (Roche, from bovine pancreas, grade II) in RPMI 1,640 supplemented with 10% (v/v) FCS. Single cell suspensions were generated by filtering over a 70 μm cell strainer. Whole tumors for histology were embedded in Tissue-Tek O.C.T. Compound (Sakura) and snap frozen in liquid nitrogen and stored at -80°C until further used. For the harvesting of the peritoneal exudate cells (PEC) the abdominal cavities were flushed with 5 mL PBS with a needle and syringe and the suspension containing PEC was extracted with the same syringe. As control, heparinized peripheral blood from healthy human donors (available through the Sanquin Blood bank or from healthy volunteers) was obtained and used according to the Declaration of Helsinki 1964.

### Human neutrophil cell isolation and immune compartment characterization

Human neutrophils from healthy human donors or from bone marrow of engrafted mice were enriched either by Percoll fractionation, as previously described (22), or by magnetic-activated cell sorting (MACS) using anti-human CD15 microbeads (Miltenyi Biotec), according to manufacturer’s instructions, respectively. The isolated neutrophils were kept in 4-(2- hydroxyethyl) -1- piperazineethanesulfonic acid supplemented with 5 g/L human albumin (Albuman; Sanquin Plasma Products), 1 mM CaCl and 5.5 mM glucose (further referred to as HEPES buffer) and were used for functional experiments.

The following directly conjugated antibodies were used for flow cytometry analysis of human cell populations in whole blood, bone marrow, spleen and tumor samples: CD45-BUV805 (clone HI30; BD Biosciences), CD19-BUV737 (clone SJ25C1; BD Biosciences), CD3-BUV661 (clone UCHT1; BD Biosciences), CD4-BUV496 (clone SK3; BD Biosciences), CD16-BUV496 (clone 3G8; BD Biosciences), Gr-1-BUV395 (clone RB6-8C5; BD Biosciences), CD15-BV605 (clone W6D3; eBioscience), CD8-BV605 (clone RPA-T8; BD BioLegend), CD25-BV421 (clone 2A3; BD Biosciences), CD11b-BV421 (clone ICRF44; BioLegend), CD11c-PerCP-Cy5.5 (clone 3.9; BioLegend), HLA-DR-FITC (clone C243; BioLegend), FoxP3-PerCP-Cy5.5 (clone 235A/E7; BD Biosciences), CD32-FITC (clone AT10, Bio-Rad), CD64-FITC (clone 10.1, Bio-Rad), CD66b-FITC (clone 80H3; Bio-Rad), CD14-PE-Cy7 (clone 61D3; eBioscience), CD56-PE (clone B159; BD Pharmingen), Siglec-8-PE (clone 7C9; BioLegend), Siglec-9-PE (clone K8; BioLegend), EMR-3-APC (clone 3D7; Bio-Rad) and CD62L-APC (DREG-56; BD Pharmingen), CD33-A700 (clone WM53; BD Biosciences). When specified, a human lineage cocktail of biotinylated antibodies (further referred as ‘Lineage’) followed by PerCP-Cy5.5-conjugated streptavidin (BD Biosciences) was used for exclusion of CD3, CD19 and CD56 populations from the analysis (Biotin-labeled anti-human CD3, clone OKT3; Biotin-labeled anti-human CD19, clone HIB9; Biotin-labeled anti-human CD56, clone CMSSB, all from eBioscience). A LIVE/DEAD Fixable Near-IR Dead Cell Stain Kit (Invitrogen) was used to exclude dead cells.

Flow cytometry data were acquired using FACS Symphony™ or Fortessa™ flow cytometer (BD Biosciences) and analyzed using FlowJo software (version 10.8; Becton Dickinson). Cell quantification was achieved by using Precision Count beads™, according to manufacturer protocol (BioLegend).

For further isolation of the neutrophil progenitors huMISTRG samples from total bone marrow of steady state animals were first enriched for CD15^+^ *via* magnetic sorting (see below) and later separated by FACS sorting based on FSC/SSC and the expression of CD11b-BV421 and CD16-PE-Cy7. FACS sorting was performed using BD FACS Aria III™ cell sorter (BD biosciences).

### Cytospin preparation and staining

0.5 or 1 × 10^5^ neutrophils were cytospun (Shandon CytoSpin II Cytocentrifuge) for 10 minutes onto 76 × 26 mm glass microscope slides (Menzel-Gläser). The cytospin slides were first air-dried and subsequently stained for 5 minutes in May-Grünwald followed by a 30 minutes staining with Giemsa. Slides were rinsed in deionized water, air-dried and analyzed with Zeiss Axio Scope A1 microscope.

### Phagocytosis assay

Phagocytic activity of FITC-labeled zymosan was assessed *via* flow cytometry. Zymosan particles (10 mg/mL; MP Biomedicals) were labeled with 0.2 mg/mL fluorescein isothiocyanate (FITC; Sigma Aldrich) for 30 minutes at 37°C in a shaker (650 rpm). The FITC-labeled particles were then opsonized with pooled serum (obtained *via* plasmapheresis from five healthy donors) for another 30 minutes to which neutrophils (0.5 × 10^6^) were added in HEPES buffer. At the desired time points, samples were added to STOPbuffer (PBS containing 20 mM sodium fluoride, 0.5% PFA and 1% BSA) and the amount of FITC fluorescence within the neutrophil gate was measured on a FACS Fortessa™ flow cytometer (BD Biosciences). Data were analyzed with FlowJo software and expressed as percentage of FITC^+^ neutrophils.

### NADPH oxidase activity assay

Nicotinamide adenine dinucleotide phosphate (NADPH)-oxidase activity was assessed by measuring the release of hydrogen peroxide (H_2_O_2_) with an Amplex Red kit (Molecular Probes). Neutrophils (0.25 × 10^6^) were left unstimulated in HEPES buffer or were stimulated for 30 minutes at 37°C with *E. coli* (OD625 = 0.2, strain ML-35), unopsonized zymosan (1 mg/mL), serum-treated zymosan (STZ, 1 mg/mL), phorbol 12-myrisatate 13-acetate (PMA, 100 ng/mL; Sigma Aldrich) or platelet-activation factor (PAF; 1 µmol/L; Sigma Aldrich)/N-formylmethionine-leucyl-phenylalanine (fMLP, 1 µmol/L; Sigma Aldrich) in the presence of Amplex Red (0.5 µmol/L) and horseradish peroxidase (1 U/mL). Fluorescence derived from Amplex Red conversion into Resorufin was measured at 30-second intervals for 30 minutes with an Infinite F200 PRO plate reader (Tecan). The activity of the NADPH oxidase of neutrophils was determined as nmol H_2_O_2_/min × 10^6^ cells.

### Dihydrorhodamine -1,2,3 flow cytometry assay

Production of intracellular ROS was analyzed *via* a flow cytometry-based DHR assay. For discrimination of neutrophil subpopulations, cells (1 × 10^6^/mL) were first pre-incubated with CD11b-BV421 and CD16-PE-Cy7 (clone 3G8, BD Pharmingen) antibodies for 20 minutes on ice in the dark. After washing, cells were mixed with 0.5 µM of DMSO-dissolved DHR (Invitrogen) for 5 minutes at 37°C in a shaker and cells were subsequently stimulated with PMA (100 ng/mL). At the desired time points, samples were added to STOPbuffer and the amount of fluorescent rhodamine-1,2,3 resulting from DHR oxidation by H_2_O_2_ was measured on a FACS Fortessa™ flow cytometer (BD Biosciences). Data were analyzed with FlowJo software and were expressed as MFI.

### Protease release measurement with DQ-BSA

Protease release after degranulation was measured with DQ-BSA (Invitrogen), which becomes fluorescent upon cleavage by proteases. Neutrophils (0.25 × 10^6^) were preincubated with cytochalasin B (CytoB, 5 µg/mL; Sigma Aldrich) for 5 minutes at 37°C in the presence of DQ-BSA (10 µg/mL) and were then stimulated with fMLP (1 µmol/L) or PMA (1 µg/mL). A 100% content value with 0.5% Triton X-100 in water was determined. Fluorescence was measured with an Infinite F200 PRO plate reader. Data were expressed as relative fluorescence units (RFU)/minute.

### Degranulation flow cytometry assay

Neutrophil degranulation was examined by preincubating the isolated cells (5 × 10^6^/mL) with the (priming) agents PAF (1 µmol/L) or CytoB (5 µg/mL) for 5 minutes and by subsequently stimulating with fMLP (1 µmol/L) for 10 minutes. Thereafter, cells were stained with directly labeled antibodies against neutrophil granule markers: CD63-APC (clone MX-49.129.5; Santa Cruz Biotechnology) or CD66b-FITC. CD11b-BV421 and CD16-PE-Cy7 antibodies were also added to the mix for the discrimination of neutrophil subpopulations. Fluorescence was measured on a FACS Fortessa™ flow cytometer (BD Biosciences) and data were analyzed with FlowJo software. Data were expressed as MFI.

### Immunohistochemistry of granule markers

Briefly, 4 x 10^4^ neutrophils from each of the flow cytometry-sorted neutrophil bone marrow progenitor were seeded on a 5 mm well of a 18 well µ-slide (Ibidi) and incubated for 30 minutes at 37°C to allow cells to attach. Cells were subsequently fixed with 4% PFA for 10 minutes and permeabilized with 0.1% Triton X-100 solution for 3 minutes. After washing, non-specific staining was reduced during blocking with PBS containing 5% BSA for 30 minutes. Cells were stained for degranulation markers with the following antibodies: unconjugated anti-human neutrophil Elastase (rabbit polyclonal; Sanquin Reagents) followed by secondary donkey anti-rabbit Alexa Fluor-555 conjugated antibody (Thermo Fisher Scientific), and biotinylated anti-human lactoferrin (goat polyclonal; Bethyl) followed by Streptavidin Alexa Fluor-647 (Invitrogen). Hoechst 33342 Solution (Thermo Fisher Scientific) was used for nuclear staining. Incubations were performed in the dark at room temperature for 45 minutes while washing with PBS in between incubations. Imaging was performed with the LSM 980 Airyscan 2 microscope (Zeiss).

### Adhesion assay

Neutrophils (5 × 10^6^/mL) were labeled with 1 µM calcein-AM (Molecular Probes) for 30 minutes at 37°C and brought to a concentration of 2 × 10^6^/mL in HEPES buffer. Calcein-labeled cells were stimulated with either PMA (100 ng/mL) or dithiothreitol (DTT, 10 mmol/L; Sigma Aldrich) in an uncoated 96-well MaxiSorp plate (Nunc) for 30 minutes at 37°C and 5% CO_2_. Cells in HEPES buffer were used to determine spontaneous adhesion. After stimulation, plates were washed with PBS to remove non-adherent cells. Adherent cells were subsequently lysed for 10 minutes at room temperature using 0.5% Triton X-100 solution in water and fluorescence was measured with a Tecan Infinite F200 PRO plate reader. Adhesion was determined as percentage of total input of calcein-labeled cells.

### Antibody-dependent cellular cytotoxicity assay

Target cells IMR-32 and NMB (1 × 10^6^), which were obtained and cultured as described previously (22), were labeled with 100 µCi 51Cr (PerkinElmer) for 90 minutes at 37°C. Chromium-labeled target cells (5 × 10^3^) were co-incubated with either unstimulated or granulocyte-macrophage colony-stimulated factor (GM-CSF, 10 ng/mL; Peptrotech) stimulated neutrophils in a 96-well U-bottom plate (Corning) in the absence or presence of dinutuximab (1 µg/mL, Unituxin, Ch14.18; United Therapeutics) in culture medium for 4 hours at 37°C and 5% CO_2_. A target:effector (T:E) ratio of 1:50 (i.e., 5.000:250.000 cells) was used. Spontaneous and maximum ^51^Cr release were determined by incubating the target cells without effector cells and by treating the target cells with a 0.1% Triton X-100 solution in culture medium, respectively. After incubation, 30 µL of supernatant was transferred to Lumaplates (PerkinElmer). The plates were dried overnight at room temperature and analyzed in a MicroBeta2 plate reader (PerkinElmer). The percentage of cytotoxicity was calculated as: [(experimental counts per minute ((CPM)−spontaneous CPM)/(maximum CPM−spontaneous CPM)]×100%. All conditions were performed in duplicate.

### Neutrophil mobilization

Mice were injected subcutaneously (S.C.) with 250 µg/kg of recombinant human granulocyte colony-stimulating factor (G-CSF, Neupogen, clinical grade, unused remains from patient treatment regimen; Amgen), 5 mg/kg CXCR4 antagonist Plerixafor (Mobizil, clinical grade, unused remains from patient treatment regimen; Sanofi/Genzyme, kindly provided by Pharmacy of Princess Maxima Center, Utrecht) or a combination of the two agents on the two consecutive days prior to sampling. Control mice were injected S.C. with sterile PBS (Gibco). On day of experiment, mice were sacrificed by carbon dioxide asphyxiation for the harvesting of peripheral blood and bone marrow samples.

### Thioglycolate-induced peritonitis model

Peritonitis was induced by a single intraperitoneal (I.P.) injection of 1 mL sterile 4% thioglycolate (Sigma Aldrich). Control mice were injected I.P. with 1 mL sterile PBS. At 16 hours after injection the mice were sacrificed and PECs were harvested from the abdominal cavities.

### Tumor model

Mel526 and NKIRTIL006 human melanoma lines were established from patient material obtained following informed consent and in accordance with local guidelines (kind gift from T. Schumacher, NKI, Amsterdam). Tumor cells were cultured in RPMI1640 supplemented with 10% FCS and penicillin (100 IU/mL) and streptomycin (100 mg/mL). 5 x 10^6^ cells in 200 mL PBS were injected S.C. in the flank of huMISTRG (level of chimerism > 20% huCD45^+^). Tumor-bearing huMISTRG animals were analyzed from 3 weeks following tumor cell engraftment.

### Immunohistochemistry of tumor samples

Frozen tumors were cut with a Leica CM1850 UV cryostat (Leica) into 10 µm thickness serial sections and subsequently collected onto SuperFrost Plus glass slides (Avantor). Prior to staining, tumor sections were fixed with 4% PFA for 10 minutes and blocked with PBS containing 0.5% BSA for 30 minutes to reduce non-specific staining. Sections were then stained for human cell populations with the following monoclonal antibodies: CD66b-BB515 (clone G10F5; BD Biosciences), purified CD3 (clone HIT3a; BioLegend) followed by secondary PE-conjugated anti-mouse antibody (Invitrogen), and Biotin-labeled CD19 (clone HIB9; eBioscience) followed by APC-conjugated streptavidin (Invitrogen). Hoechst 33342 Solution (Thermo Fisher Scientific) was used for nuclear staining. Incubations were performed in the dark at room temperature for 45 minutes, using Tris-Buffered Saline with 0.1% Tween-20 detergent for washes between incubation steps. Sections were subsequently mounted with 10% Mowiol supplemented with 2.5% DABCO and analyzed with the Nikon Ti2e microscope (Leica Microsystems). A Tilescan of the entire tumor was taken with Kinetix sCMOS camera (objective 10x; Photometrics). Files were first Denoised using the Algorithm provided by Nikon and subsequently processed with a rolling ball filter (14.86 µm). Crops were taken from the Tilescans.

### Statistical analysis

Statistical analysis was performed with GraphPad Prism version 9 (GraphPad Software). Data were evaluated by one-way or two-way ANOVA, and, where indicated, correction for multiple comparisons using either Sidak’s or Tukey’s test was performed, or paired two-tailed student’s t-test. The results are presented as the mean ± SEM. Data were considered significant when p < 0.05.

## Results

### General characterization of human immune compartments, including human neutrophils, after reconstitution of huMISTRG mice

To study the level of human immune reconstitution in huMISTRG mice (generated as shown in **Figure 1A**), we studied the kinetics of the humanization procedure by determining the percentage of human CD45^+^ cells in peripheral blood of the mice at different timepoints after CD34^+^ cell engraftment (**Figure 1B**). As early as 8 weeks post-injection, >50% human CD45^+^ cells were detected in blood, and these levels steadily increased over time, reaching up to ~70-90% of human CD45^+^ cells 10 to 14 weeks from transplantation. We characterized adult huMISTRG for multilineage immune cell differentiation and, consistent with other literature in huMISTRG animals (6), we found all major immune compartments represented in spleen, peripheral blood and bone marrow (**Figure 1C**). This included T (CD3^+^) and B lymphocytes (CD19^+^), NK cells (CD56^+^), myeloid cells (CD33^+^), and dendritic cells (CD11c^+^HLA-DR^+^).

**Figure 2:**
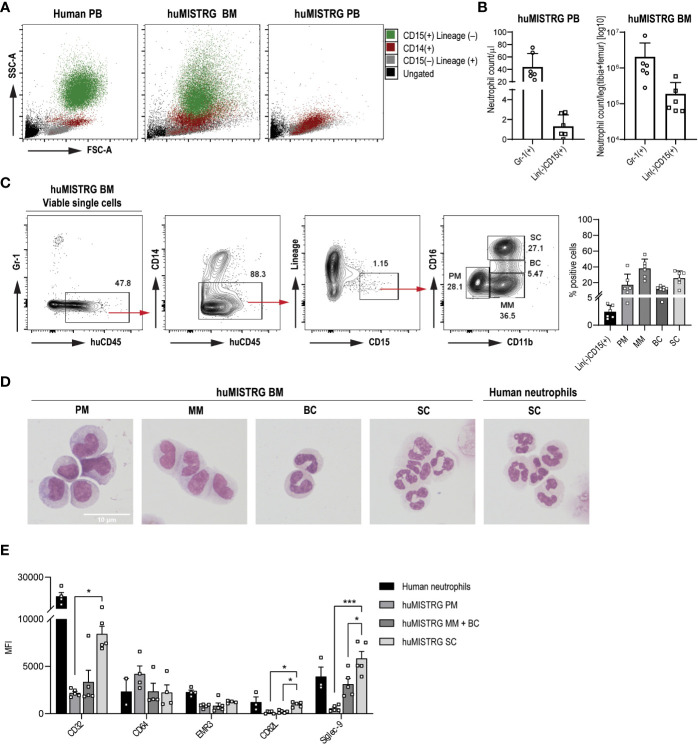
Human neutrophil development and phenotype in huMISTRG mice **(A)** SSC-A vs FSC-A density plots for the identification of the different immune cell populations of human PB, huMISTRG BM or huMISTRG PB: ungated (black), CD15^–^Lineage^+^ (human T, B and NK cells, grey), CD14^+^ (human monocytes, red), CD15^+^Lineage^–^ (human neutrophils, green). **(B)** Quantification of Gr-1^+^ murine, and Lineage^–^CD15^+^ human neutrophils in bone marrow, and in peripheral blood of huMISTRG animals at steady state. N=2-4, of 2 independent experiments. **(C)** Representative sequential gating for identification of BM human neutrophils (huCD45^+^CD14^–^Lineage^–^CD15^+^) and subpopulations (CD11b vs CD16; PM, MM, BC, SC) in huMISTRG mice, gated on viable single cells. Human eosinophils (Siglec-8^+^), being <0.2% of the whole sample, were also excluded from the gating (not shown). Numbers indicate the percentages of the gated populations. Percentage of positive cells from BM human neutrophils as Lineage^–^CD15^+^ and subpopulations gated as in gating strategy on the left is shown for n=6, of 4 individual experiments (right). **(D)** Representative cytospins of each neutrophil progenitor (PM, MM, BC, SC) from magnetically enriched CD15^+^ huMISTRG BM fraction after flow cytometry sorting based on CD11b and CD16 expression after May-Giemsa staining (objective 100x). Human neutrophils from peripheral blood were used as control for comparison of end-stage segmented nucleus. **(E)** Neutrophil marker expression (CD32, CD64, EMR3, CD62L and Siglec-9) on the different huMISTRG neutrophil subpopulations (PM, MM+BC, SC) and on human neutrophils. N=2-5. PM, promyelocytes; MM, metamyelocytes; BC, band cells; SC, segmented cells; MFI, mean fluorescence intensity; FSC-A, forward scatter-area; SSC-A, side scatter-area; PB, peripheral blood; BM, bone marrow. * = p < 0.05, *** = p < 0.001.

Importantly, in accordance with previous findings (6, 16), we also found low level of human neutrophils in peripheral blood of huMISTRG mice at steady state (**Figures 2A, B** **)**, identified as huCD45^+^Gr-1^–^CD14^–^CD15^+^Lineage^–^ (see gating strategy on **Figure 2C**), while these were amply represented in bone marrow (**Figures 2A, B** **)**. To discriminate between the four different neutrophil developmental stages existing in the bone marrow niche, namely promyelocytes (PM), metamyelocytes (MM), band cells (BC) and segmented cells (SC) (23), we performed flow cytometry analysis based on expression of cell surface markers CD11b and CD16 on the (human) CD15^+^ population (**Figure 2C**) **(** 24–26). This allowed us to confirm the presence of all neutrophil maturation stages from CD11b^–^CD16^–^ PM to end-stage CD11b^+^CD16^+^ SC in huMISTRG bone marrow samples, both phenotypically and morphologically (**Figures 2C, D** **)**. A morphological analysis of the nucleus of each of the different FACS sorted subpopulations confirmed that the neutrophil progenitors from bone marrow of huMISTRG mice very closely resembled those that are found in human bone marrow, with the more immature cells showing a rounder and more banded nucleus, and the more mature ones already showing a number of nuclear segmentations (**Figure 2D**) **(** 24). When specifically analyzing the human neutrophil phenotype, we found that huMISTRG neutrophils from bone marrow acquired an elevated expression level of Fc gamma receptor CD32 (Fcγ receptor IIa), activation marker CD62L (L-selectin), and differentiation marker Siglec-9 upon maturation, with the most mature subset showing a similar phenotype as circulating neutrophils from human blood. No significant differences between neutrophil subpopulations were seen for Fc gamma receptor CD64 (Fcγ receptor I) and maturation marker EGF-like module-containing mucin-like hormone receptor-like 3 (EMR3, **Figure 2E**).

Together, huMISTRG mice displayed multilineage human immune reconstitution with representation of the myeloid compartment, including dendritic cells, monocytes and neutrophils. Irrespective of the minimal neutrophil numbers in blood of huMISTRG animals in steady state, we confirmed the presence of all human neutrophil maturation stages in huMISTRG bone marrow, similar to those described in human bone marrow (24).

### Bone marrow neutrophils of huMISTRG mice show close to physiological *ex vivo* functionality

**Figure 3:**
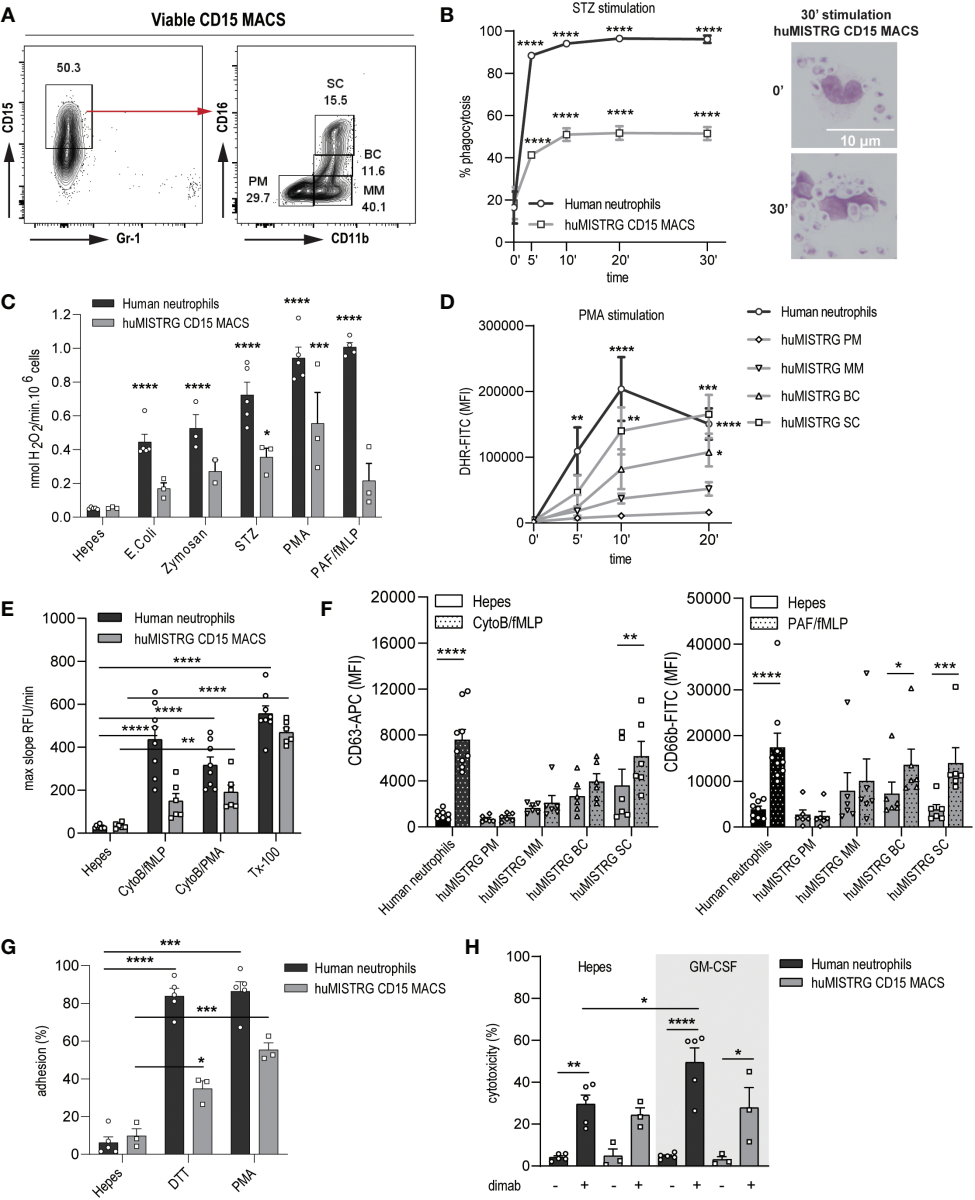
Close to physiological *ex vivo* functionality by neutrophils from bone marrow of huMISTRG mice. **(A)** Gating strategy of viable CD15 MACS-sorted cells of huMISTRG mice showing enrichment after sorting and human neutrophil BM subpopulations based on CD11b and CD16 staining (PM, MM, BC, SC). Numbers indicate the percentages of the different populations. **(B)** Phagocytosis of FITC-labeled serum-opsonized (STZ) zymosan particles from 0 to 30 min by human neutrophils (black line) and CD15 MACS-sorted huMISTRG neutrophils (grey line) as assessed by flow cytometry. N= 3, of two individual experiments. Statistical differences compared to respective unstimulated condition. Representative microscopic images from cytospin slides (right) after May-Giemsa staining (objective 50x) at timepoint 0 and 30 min depicting zymosan particles inside the neutrophil only at 30 min timepoint. **(C)** NADPH-oxidase activity of human neutrophils (black bars) and CD15 MACS-sorted huMISTRG neutrophils (grey bars) in the presence of the indicated stimuli expressed as nmol H_2_O_2_/min per 10^6^ cells. N=2-5, of three individual experiments. Statistical differences compared to respective Hepes condition. **(D)** PMA-induced DHR oxidation from 0 to 20 min by mature human neutrophils (black line) and from the different neutrophil BM subpopulations based on CD11b and CD16 gating (PM, MM, BC, SC) within the neutrophil-characteristic FSC/SSC pattern of huMISTRG mice (grey lines). N=3-5, of three individual experiments. Statistical differences compared to respective unstimulated condition. **(E)** Protease activity of human neutrophils (black bars) and CD15 MACS-sorted huMISTRG neutrophils (grey bars) in the presence of the indicated stimuli, or Triton (Tx-100) for total release, expressed as max slope RFU/min. N=6-9, of six individual experiments. **(F)** Surface exposure of CD63 (azurophilic granules) and CD66b (specific granules) upon stimulation (dotted bars) with CytoB/fMLP and PAF/fMLP, respectively, on mature human neutrophils (black bars) and on the different neutrophil BM subpopulations based on CD11b and CD16 gating (PM, MM, BC, SC) within the neutrophil-characteristic FSC/SSC pattern of huMISTRG mice (grey bars). N=6-9, of six individual experiments. **(G)** Evaluation of adhesion capacity in the presence of the indicated stimuli on human neutrophils (black bars) and CD15 MACS-sorted huMISTRG neutrophils (grey bars) as determined by the percentage of total input of calcein-labeled cells. N=3-5, of three individual experiments. **(H)** ADCC of IMR-32 neuroblastoma cells opsonized with (+) or without (−) dinutuximab (dimab) by unstimulated (Hepes) or GM-CSF stimulated human neutrophils (black bars) and CD15 MACS-sorted huMISTRG neutrophils (grey bars). N=3-5, of three individual experiments. PM, promyelocytes; MM, metamyelocytes; BC, band cells; SC, segmented cells; RFU, relative fluorescent units; DHR, dihydrorhodamine; MFI, mean fluorescence intensity; ADCC, antibody-dependent cellular cytotoxicity. * = p < 0.05; ** = p < 0.01; *** = p < 0.001; **** = p < 0.0001.

In order to study the functionality of human neutrophils in our model system, we isolated this population from bone marrow of huMISTRG mice by MACS using anti-human CD15 microbeads. This led to an enrichment of ~50% of CD15^+^ MACS-sorted cells with still some CD15^low^ cells present, consisting mainly of CD14^+^ monocytes (**Figure 3A** and **Suppl. Figures 1A, B** **)**. Despite lacking cells of the murine adaptive immune system (6), huMISTRG animals still had a considerate amount of Gr-1^+^ murine neutrophils, but these were excluded completely by CD15-based MACS purification (**Suppl. Figures 1C, D** **)**. Within the CD15^+^ MACS-sorted samples, all four known neutrophil bone marrow subpopulations, as defined by expression of CD11b and CD16, were represented (**Figure 3A** and **Suppl. Figure 1B**) **(** 24). This cell suspension consisting of CD15^low^ and CD15^+^ cells, containing both immature and mature neutrophils, was the population used for subsequent functional studies.

Neutrophils are endowed with the unique capacity to engulf and subsequently kill invading microbes through phagocytosis, which is essential for the maintenance of host health (27). Hence, we first investigated the huMISTRG neutrophil’s ability to ingest fluorescently-labeled serum-opsonized zymosan (STZ) *via* a FACS-based phagocytosis assay. We observed that neutrophils from huMISTRG animals significantly phagocytosed FITC-conjugated zymosan particles reaching levels of 50% as early as 10 minutes post-stimulation, at which point plateau was achieved (**Figure 3B**). A similar pattern was found for human control neutrophils, although these reached higher levels of phagocytosis (>90%), likely explained by the different proportions of immature cells in these samples (**Suppl. Figure 1B**). Cytospin analysis of the samples confirmed the presence of zymosan particles overloading the neutrophil’s cytoplasm at the latest timepoint, indicative of efficient phagocytosis by huMISTRG CD15-sorted samples.

Neutrophils eliminate bacteria or other pathogens by the release of highly toxic ROS *via* the NADPH oxidase system (28, 29). We used an Amplex Red hydrogen peroxide assay to assess the ability of neutrophils to respond to a number of microbial stimuli. As expected, *E. coli*, zymosan, STZ, phorbol 12-myrisatate 13-acetate (PMA) and platelet-activation factor/N-formylmethionine-leucyl-phenylalanine (PAF/fMLP) were potent inducers of ROS in human control neutrophils (**Figure 3C**). huMISTRG neutrophils were also found to respond to all stimuli tested in a similar trend, indicative of a functional NADPH oxidase complex in the overall population of MACS-sorted bone marrow samples. To assess the ability of each of the neutrophil bone marrow progenitors from our samples to produce intracellular ROS, we performed a flow cytometry-based assay with prior staining for CD11b and CD16 (**Figure 3D**). The capacity of huMISTRG neutrophils to generate ROS in response to PMA stimulation positively correlated with the maturation state of the cell, with the segmented cells fraction outperforming all other less mature fractions, as would also be expected for neutrophils that develop in the human bone marrow (24), and being comparable to that of mature control neutrophils. As the end-stage segmented cells make up only 15-30% of the CD15^+^ population used in these assays (**Figure 3A**), this likely explains the lower overall ROS production by the unfractionated population (than by human control neutrophils) tested in **Figure 3C**.

As neutrophils use degranulation of proteolytic enzymes to combat infections (30–33), we examined the presence of granule-related proteins for azurophilic (neutrophil elastase) and specific (lactoferrin) granules within the different BM neutrophil precursors by confocal imaging, and confirmed that they are indeed contained within their cytoplasm in steady state (**Suppl. Figure 2A**) **(** 26, 34). Next, we assessed the proteolytic ability of huMISTRG neutrophils by a DQ-BSA assay. Upon full lysis with triton (Tx-100), both human and huMISTRG neutrophil populations successfully cleaved the DQ-BSA substrate, resulting in fluorescence, performed by the potent hydrolytic enzyme neutrophil elastase, among others (**Figure 3E**). Since we performed the assay using total CD15^+^ MACS-sorted fractions, the number of mature neutrophils within our huMISTRG samples only sufficed to detect a significant DQ-BSA cleavage in response to CytoB/PMA, while significance was not achieved for CytoB/fMLP. However, when directly measuring the surface expression of CD63 and CD66b in the fractionated huMISTRG bone marrow samples thanks to prior staining for CD11b and CD16, we detected upregulation of both azurophilic (CD63) and specific (CD66b) granule markers in response to adequate activation from band cell stage onward, coinciding with initiation of FPR1 expression (fMLP receptor, **Figure 3F**) **(** 26, 31, 34, 35). Once again, this suggests that the reduced response of the entire huMISTRG neutrophil population (as compared to that of human control neutrophils) to the same stimulus, when measured by the DQ-BSA assay, is explained by the relatively low proportion of end-stage segmented cells, which are the only cells exhibiting clear degranulation capacity (**Figure 3E, F** **)**. Of note, in a parallel experiment we found that only neutrophils had proteolytic capabilities while monocytes had none (**Suppl. Figure 2B**), suggesting that only the CD15^+^ neutrophil population, and not the CD14^+^ cells, within the CD15-sorted samples was responsible for the protease activity that was measured.

Neutrophil adhesion is important for the extravasation into inflamed tissues (36, 37), which is a process dependent on CD11b/CD18 integrin (38, 39). huMISTRG neutrophils strongly adhered to plastic in response to both outside-in (DTT) and inside-out integrin (PMA) activation-dependent stimuli, suggesting that integrin function was fully operational in these cells (**Figure 3G**). Neutrophils can have a dual role within the tumor microenvironment, having pro-tumor activity as myeloid-suppressor cells or anti-tumor activity by ADCC (18, 19, 40, 41). As a read-out of anti-tumor ADCC, we assessed whether huMISTRG neutrophils could kill antibody-opsonized tumor cells. We co-incubated either unstimulated or GM-CSF stimulated neutrophils with neuroblastoma cell lines IMR-32 and NMB in the presence or absence of the therapeutic antibody dinutuximab, which binds to GD2, a target on this tumor type (**Figure 3H** and **Suppl. Figure 2C**). huMISTRG neutrophils were able to induce cytotoxicity towards dinutuximab-opsonized neuroblastoma cells within the 4-hour co-incubation with tumor cells. They did so less efficiently than human blood-derived neutrophils (**Figure 3H** and **Suppl. Figure 2C**, grey bars). This may at least partially be explained by the fact that the huMISTRG neutrophil population contained a mixture of mature and immature neutrophils, unlike the human blood derived cells, which were all mature. Of note, the possibility that the monocyte population still present within our MACS-sorted samples contributed to this effect was minimal as monocytes are known to require longer (overnight) incubation times to induce efficient cytotoxicity (42–44).

Overall, the huMISTRG bone marrow-derived, CD15 MACS-sorted neutrophils exhibited close to physiological *ex vivo* functionality, which correlated with the maturation state of the cells.

### *In vivo* human neutrophil migration to the periphery and peritoneum in response to mobilizing agents and inflammation

**Figure 4:**
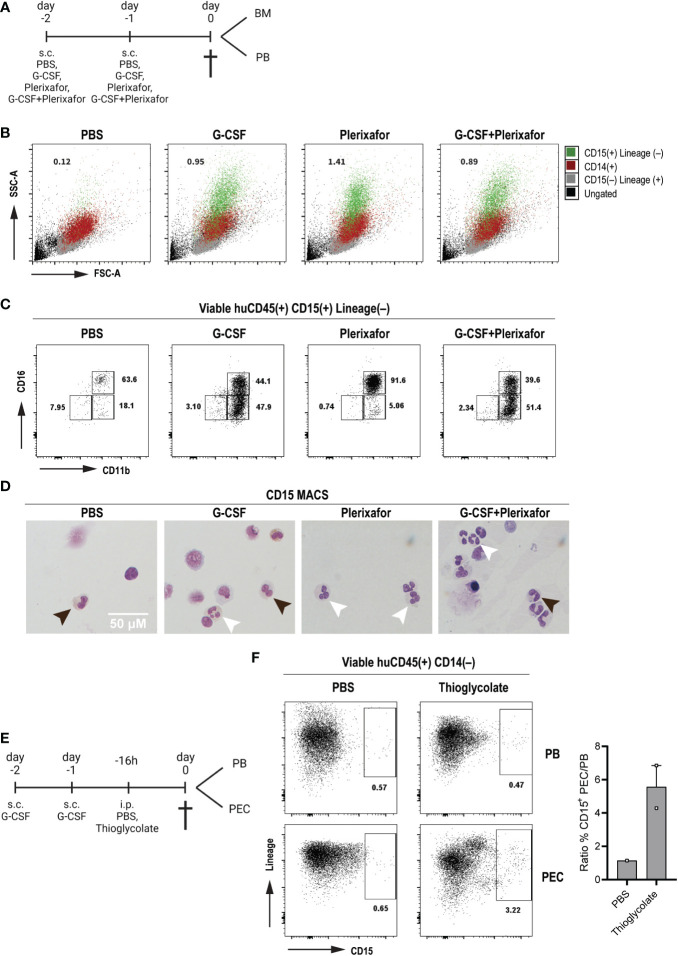
*In vivo* human neutrophil migration to the periphery and peritoneum in response to mobilizing agents and inflammation **(A)** Schematic representation of the treatment scheme with mobilizing agents until tissue sampling. Created with BioRender.com. **(B)** SSC-A vs FSC-A density plots for the identification of the different immune cell populations of mobilized PB huMISTRG samples gated as viable huCD45^+^: ungated (black), CD15^–^Lineage^+^ (human T, B and NK cells, grey), CD14^+^ (human monocytes, red), CD15^+^Lineage^–^ (human neutrophils, green). Numbers indicate the percentages of mobilized human neutrophils as CD15^+^Lineage^–^. **(C)** Representative CD11b vs CD16 flow cytometry plots of mobilization of PB huMISTRG neutrophils in response to different treatments, gated on viable human CD45^+^CD15^+^Lineage^–^ cells. Numbers indicate the percentages of the different subpopulations. **(D)** Representative cytospins of mobilized CD15-MACSed PB huMISTRG neutrophils for each respective treatment condition after May-Giemsa staining (objective 50x). Black arrows indicate less mature neutrophil with round/banded nucleus, white arrows indicate mature neutrophil with segmented nucleus. **(E)** Schematic representation of the treatment scheme with G-CSF and thioglycolate until tissue sampling. Created with BioRender.com. **(F)** Representative Lineage vs CD15 flow cytometry plots of G-CSF mobilized mice in response to peritoneal injection of thioglycolate, gated on viable human CD45^+^CD14^–^ cells. Numbers indicate the percentages of the different subpopulations. On the right, quantification of the influx of neutrophils in the peritoneum per condition represented by the ratio of CD15^+^ cells in the PEC suspension to those in the peripheral blood. N=1-2, of two individual experiments. PEC, peritoneal exudate cells; PB, peripheral blood; FSC-A, forward scatter-area; SSC-A, side scatter-area.

Given the low number of circulating human neutrophils in steady state conditions in huMISTRG animals, we sought to determine whether huMISTRG neutrophils could be mobilized from bone marrow into the periphery. To do so, adult huMISTRG mice were treated with two well-established neutrophil mobilizing agents: G-CSF (Neupogen) and the CXCR4 antagonist Plerixafor (Mobizil) (45–47), which were administered as single agents or in combination for two consecutive days prior to analysis (**Figure 4A**). We found a pronounced mobilization of neutrophils (assessed by the appearance of CD15^+^ cells with a characteristic FSC/SSC pattern) into peripheral blood in response to all treatment conditions, while no circulating neutrophils were detected in the control group (**Figure 4B**). Plerixafor treatment as a single agent induced the mobilization of mainly end-stage CD11b^+^CD16^+^ neutrophils, also confirmed microscopically by the presence of at least 3 nuclear lobes, in accordance with the morphology of mature human neutrophils (**Figures 4C, D** **) (** 23). On the other hand, treatment with G-CSF alone or in combination with Plerixafor mobilized both CD11b^+^CD16^–^ and CD11b^+^CD16^+^ neutrophils, and these cells correspondingly exhibited either a more banded nucleus or a multilobulated nucleus, respectively (**Figures 4C, D** **)**. Of note, the end-stage CD11b^+^CD16^+^ neutrophil population was absent in the bone marrow compartments of the same mobilized mice, especially for mice treated with G-CSF as a single agent or in combination with Plerixafor (**Suppl. Figure 3A**). Importantly, it was difficult to assess the effect of the mobilizing agents on the murine neutrophils (Gr-1^+^) in huMISTRG animals, since the vast majority of these were already circulating under steady-conditions (**Suppl. Figure 3B**). Yet, a slight decrease of Gr-1^+^ cells was observed in the bone marrow compartment after treatment, suggesting that a small pool of murine neutrophils did mobilize to the periphery in response to G-CSF and/or Plerixafor. The fact that murine neutrophils responded to human mobilizing agents was not unexpected, since complete cross-reactivity exists between human and mouse G-CSF (48).

To investigate whether the human neutrophils in huMISTRG mice could respond to inflammation *in vivo*, we subjected the mice to thioglycolate-induced acute sterile peritoneal inflammation following neutrophil mobilization with G-CSF (**Figure 4E**). This is a commonly used approach to study the development of an inflammatory reaction in mice due to the simple isolation of peritoneal exudate cells (PEC), with neutrophils being the first cells recruited to the injection site (49, 50). Flow cytometric assessment of the PEC suspension allowed us to determine the composition of the infiltrated cell population. Although we found murine neutrophils as Gr-1^+^ cells to strongly respond to thioglycolate (**Suppl. Figure 3C**), an influx (6-fold increase) of human neutrophils as CD15^+^ cells within the PEC suspension were also observed as compared to the control group (**Figure 4F**), indicative that the human neutrophils in huMISTRG mice have the ability to migrate towards inflammatory chemokines *in vivo*.

Taken together, not only were the human bone marrow neutrophils released into peripheral blood in response to mobilizing agents, but they also effectively responded to a local (peritoneum) sterile inflammation, suggestive of their adequate migration capacities *in vivo*.

### Human immune response with neutrophil infiltration in tumors engrafted in huMISTRG mice

**Figure 5:**
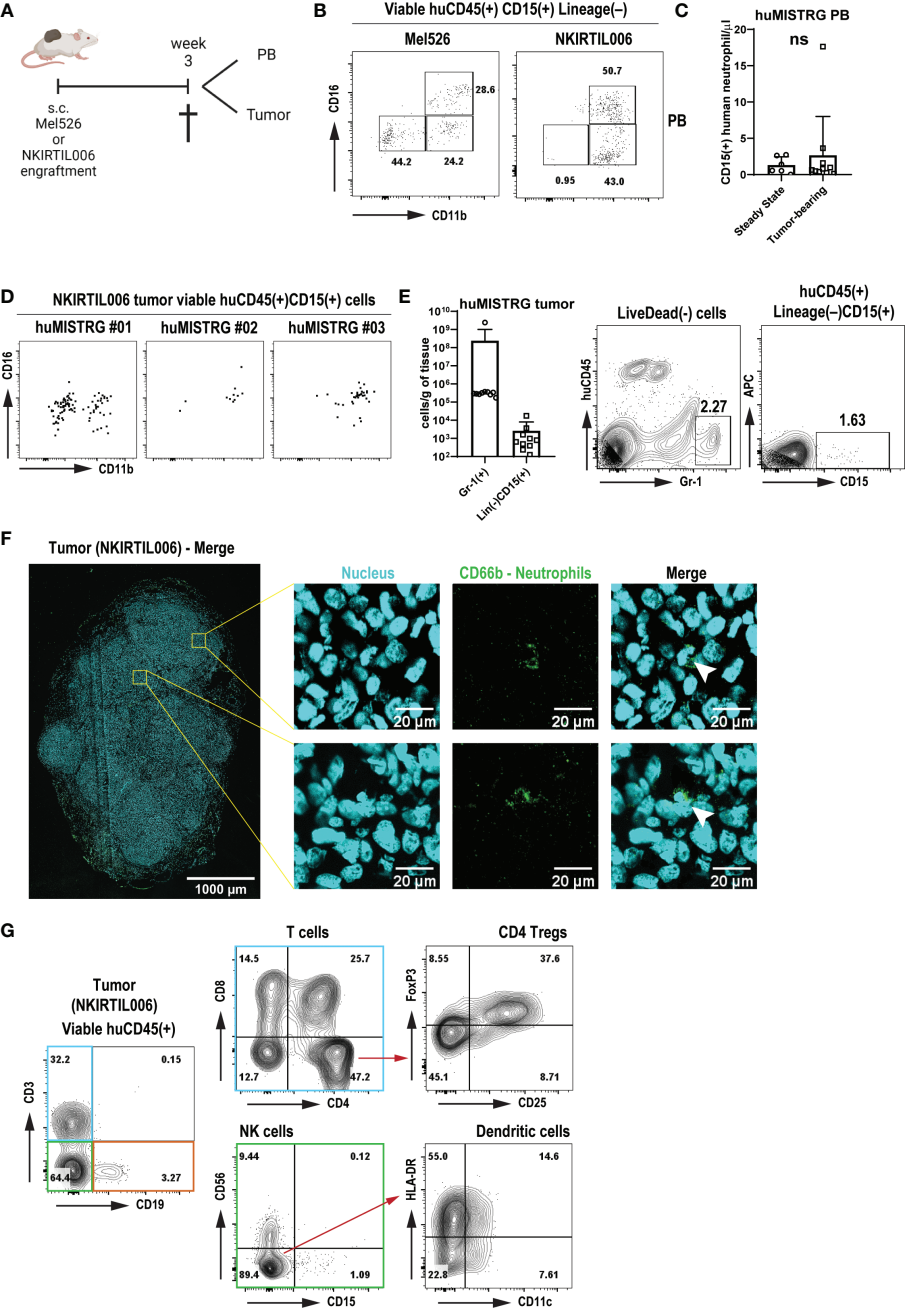
Human immune response with neutrophil infiltration in tumors engrafted in huMISTRG mice **(A)** Schematic representation of the tumor cell engraftment of the patient-derived Mel526 and NKIRTIL006 melanoma cells into the flank of adult huMISTRG mice until tissue sampling. Created with BioRender.com. **(B)** Representative CD11b vs CD16 flow cytometry plots of blood-circulating neutrophils in Mel526 (left) and NKIRTIL006 (right) tumor-bearing huMISTRG mice. Numbers indicate the percentages of the different populations. **(C)** Quantification of blood-circulating CD15^+^ human neutrophils at steady state, and in tumor-bearing huMISTRG animals. N=6-10, of 2 independent experiments. **(D)** Representative CD11b vs CD16 flow cytometry plots of three different NKIRTIL006 tumor-bearing mice, gated on viable human CD45^+^CD3^–^CD19^–^CD56^–^CD68^–^CD15^+^CD14^–^ cells. **(E)** Quantification of intratumoral Gr-1^+^ murine, and CD15^+^ human neutrophils in tumor-bearing huMISTRG animals (left). Representative flow cytometry plots of intratumoral Gr-1^+^ murine, and CD15^+^ human neutrophils (right). N=10, of 2 independent experiments. **(F)** Representative wide-field fluorescent image of a 10 µm NKIRTIL006 tumor section stained for human neutrophils (CD66b, green) and nuclear marker (Hoechst, cyan). Crops (right) were taken from the indicated Tilescan areas of the entire tumor. White arrows indicate CD66b positive staining surrounding a banded nucleus, characteristic nuclear morphology of human neutrophils. **(G)** Representative flow cytometry plots of the human immune infiltration in the tumor tissue of NKIRTIL006 tumor-bearing mice, distinguishing T cells (blue gate) from all other immune cells (green and orange gates), gated on viable human CD45^+^ cells. Numbers indicate the percentages of the different populations. PB, peripheral blood. ns, not significant.

As above-mentioned, in addition to their roles in inflammation and infection, neutrophils are increasingly recognized as critical players during cancer progression, where they can acquire either immunosuppressive functions (pro-tumor activity) or contribute to tumor elimination through ADCC (anti-tumor activity) (18, 19). To determine whether huMISTRG animals can be used to study human intratumoral neutrophils, we engrafted huMISTRG mice with human tumors. We used patient-derived human melanoma cell lines Mel526 and NKIRTIL006 as tumor models (51), which were subcutaneously injected in the flank of adult huMISTRG mice. Tumors grew gradually for up to 3 weeks at which point peripheral blood and tumors were harvested for analysis (**Figure 5A**).

We detected blood-circulating human neutrophils of both CD11b^+^CD16^–^ and CD11b^+^CD16^+^ phenotype in both tumor models (**Figure 5B**), although these were not significantly higher numbers than circulating human neutrophils detected at steady state, as shown by absolute cell count (**Figure 5C**). Notably, tumor engraftment was also able to mobilize murine Gr-1^+^ neutrophils, as shown by the increased number of both blood-circulating and tumor-infiltrating murine neutrophils (**Figure 5E** and **Suppl. Figures 4A, B**). This result suggests that a chronic inflammatory setting solely generated by the presence of a tumor was sufficient to mobilize human neutrophils from bone marrow into tumor tissue. Strikingly, in addition to murine neutrophils (**Suppl. Figures 4A, B** **)**, human neutrophils were found to infiltrate NKIRTIL006 tumors isolated from huMISTRG animals as detected by CD15^+^ staining with flow cytometry analysis, which were further characterized for maturation markers CD11b and CD16 (**Figures 5D, E** **)**. In addition, analysis of the entire tumor tissue *via* wide-field fluorescence microscopy further confirmed the presence of intratumoral neutrophils as depicted by positive staining for CD66b surrounding a banded nucleus characteristic of human neutrophils (**Figure 5F**). In fact, the human neutrophils were found in a tumor environment that contained a complete human immune infiltrate consisting of a lymphoid compartment of B cells (CD19^+^), NK cells (CD56^+^) and T cells (CD3^+^) (**Figure 5G** and **Suppl. Figure 4C**), with CD4^+^ (including regulatory T cells CD25^+^FoxP3^+^) and CD8^+^ T cells, and a subset of CD11c^+^HLA-DR^+^ human dendritic cells, recapitulating the immune landscape in patients (52, 53).

In summary, these results show that huMISTRG mice develop a human immune infiltrate in melanoma tumors, and potentially provide a model to study responses of human neutrophils in human solid tumors.

## Discussion

Mice transplanted with a human hemato-lymphoid system aim to help close the gap for translating the findings derived from rodents to humans (54). The development of the neutrophil lineage – the most abundant circulating leukocyte and the first line of defense against infections in humans (17) – in such mice remains defective in traditional humanized mouse strains (54). In this study, we investigated the functionality of the human neutrophil population in huMISTRG mice, a strain that allows reconstitution of a much more complete human immune system than previous models (6). We demonstrate that this model system can be suitable for the study of neutrophil biology in human immune processes.

Previous work with huMISTRG mice has shown the presence of only a small number of human neutrophils in the peripheral blood of these mice at steady state, while these are abundantly present in the bone marrow (6). Rongvaux et al. suggested that the terminal differentiation of neutrophils in this mouse environment may be suboptimal, as seen for other humanized strains such as *NSG-SGM3* – NSG mice engineered to constitutively express human stem cell factor, GM-CSF and IL-3 cytokines –, where these displayed the morphology and cell surface phenotype of immature cells (16, 55). In contrast, we found that huMISTRG mice do generate end-stage neutrophils with a CD11b^+^CD16^+^ phenotype and a segmented nucleus, which possess functional capacity in the bone marrow. Others suggested that the egress of neutrophils from the bone marrow could be impaired (6, 54) due to incompatibilities in adhesion molecules and chemokine-chemokine receptor pairs between species (56, 57). However, treatment with the CXCR4 antagonist Plerixafor or with human G-CSF resulted in the release of end-stage human neutrophils into the circulation of huMISTRG animals. This shows that at least the SDF-1α/CXCR4 chemokine axis apparently operates across species and that also the adhesion steps required for migration out of the bone marrow are functional. The dearth of human neutrophils in the circulation of huMISTRG mice at steady state might be explained by a lack of chemotactic cues to induce egress of mature neutrophils from the bone marrow in the absence of inflammation or infections. Indeed, huMISTRG animals are housed in exceptionally clean environments under specific-pathogen-free (SPF) conditions and are maintained under continuous prophylactic large spectrum antibiotics treatment, which altogether may contribute to the containment of the mature human neutrophil pool in the bone marrow niche. It is important to note in this regard that even regular SPF laboratory mice have markedly more neutropenic blood than humans (1, 58).

We have shown that the huMISTRG model is amenable to studying antimicrobial properties of human neutrophils. In particular, we demonstrated neutrophil-specific responses towards physiological antimicrobial stimuli. *In vivo*, the extravasation of human neutrophils into the peritoneal cavity of huMISTRG mice after thioglycolate injection further demonstrated the potential to study (trans)migration capacities of neutrophils triggered by chemotactic cues (49, 59). The huMISTRG model could thus be used to test other qualitative human neutrophil functions in various *in vivo* experimental set ups, such as cecal ligation and puncture, ischemia-reperfusion injury, LPS nebulization, sterile heart injury, laser injury in skin or cremaster (60). Moreover, the discovery of circulating and intratumoral human mature neutrophils in tumor-bearing mice suggests that huMISTRG animals can also be used to address neutrophil functions in cancer *in vivo*. Despite de fact that the tumor microenvironment is mostly of murine origin (*i.e.*, endothelium, extracellular matrix, fibroblasts), human neutrophils are evidently still able to extravasate in response to mobilizing factors (such as G-CSF (61)) produced by the human tumor engrafted in huMISTRG mice. There is debate on the nature and function of infiltrated neutrophils in human tumors (62). On the one hand, neutrophils may promote tumor growth as myeloid-derived suppressor cells, while on the other, they have been implicated as effectors (via ADCC) of antibody treatment of cancer, such with the use of dinutuximab in neuroblastoma patients (22, 63). The huMISTRG model could be further developed into a tool for obtaining the evidence needed to clarify their role.

As described previously (6), huMISTRG display graft-versus-host disease-related erythropenia, that ultimately leads to severe anemia. In our hands, this was not a limiting factor in our study, which involved relatively short term experiments (up to 20 weeks after birth). In addition, breeding of MISTRG animals was similar to other strains. Nevertheless, one should consider such limitations in the case of long-term experiments. One drawback of the MISTRG mouse model remains that the number of human neutrophils is low. A recent study showed that greater numbers of blood circulating human neutrophils can be obtained in MISTRG mice by replacing the murine gene encoding G-CSF with the human version, in combination with deletion of the murine G-CSF receptor gene, which may partially be explained by the elimination of competition between murine and human neutrophils (64). As tested in that study, it seems likely that the neutrophils in that model are functional and capable of intra- and extravasation, just as we have shown here for “regular” MISTRG mice. In these latter mice, competition with endogenous murine neutrophils could be eliminated *via* injection of anti-Gr-1 depleting antibodies which is known to lead to a profound and durable neutropenia (65). Taken together, although the presence of murine neutrophil in such experimental design should be considered, our data do show that the huMISTRG provides a potential model system for the study of neutrophil biology in complex human diseases, such as the preclinical evaluation of their responses to novel immunotherapeutic approaches against solid cancer and for testing the role of genetic backgrounds or manipulations on their function *in vivo.*

## Data availability statement

The original contributions presented in the study are included in the article/**Supplementary Material**. Further inquiries can be directed to the corresponding authors.

## Ethics statement

Mel526 and NKIRTIL006 human melanoma lines were established from patient material obtained following informed consent and in accordance with local guidelines (kind gift from T. Schumacher, NKI, Amsterdam). Human blood samples were obtained from anonymized volunteers with written informed consent with approval from the Medical Ethical Committee of Sanquin Research and Landsteiner Laboratory in accordance with the Declaration of Helsinki.

## Author contributions

PM-S and JK designed and performed the experiments, analyzed the data and wrote the manuscript, with the help of DA. MH, AL, ES and NB performed experiments and reviewed the manuscript. GT helped with the study design and reviewed the manuscript. DA, RF, AR, and RB contributed to data interpretation and reviewed the manuscript. TK, HM and KF designed the experiments, interpreted and evaluated the data and reviewed the manuscript. All authors contributed to the article and approved the submitted version.

## Glossary

**Table:**

ADCC	antibody-dependent cellular cytotoxicity
ANOVA	analysis of variance
BC	band cells
CB	cord blood
CytoB	cytochalasin B
BSA	bovine serum albumin
BM	bone marrow
CPM	counts per minute
DHR	dihydrorhodamine
Dimab	dinutuximab
DMSO	dimethyl sulfoxide
DTT	dithiothreitol
EDTA	ethylenediaminetetraacetic acid
EMR-3	EGF-like module-containing mucin-like hormone receptor-like 3
FACS	fluorescence-activated cell sorting
FCS	fetal calf serum
fMLP	N-formylmethionyl-leucyl-phenylalanine
FSC	forward scatter
G-CSF	granulocyte colony-stimulating factor
GD2	disialoganglioside
GM-CSF	granulocyte-macrophage colony-stimulating factor
HEPES	4-(2- hydroxyethyl) -1- piperazineethanesulfonic acid
HLA-DR	human leukocyte antigen-DR isotype
HPC	hematopoietic progenitor cell
huMISTRG	humanized MISTRG
I.P.	intraperitoneal
MACS	magnetic-activated cell sorting
M-CSF	macrophage colony-stimulating factor
MFI	mean fluorescence intensity
MISTRG	M-CSF^h/h^ IL-3/GM-CSF^h/h^ SIRPa^h/h^ TPO^h/h^ RAG2^–/–^ IL2Rγ^–/–^
MM	metamyelocytes
NADPH	nicotinamide adenine dinucleotide phosphate
NK	natural killer
NSG	NOD/scid/IL2Rγ^–/–^
NOD scid gamma PAF	platelet-activation factor
PB	peripheral blood
PBMC	peripheral blood mononuclear cells
PBS	phosphate buffered saline
PEC	peritoneal exudate cell
PFA	paraformaldehyde
PM	promyelocytes
PMA	phorbol 12-myrisatate 13-acetate
RFU	relative fluorescence unit
ROS	reactive oxygen species
RPMI	Roswell Park Memorial Institute’s medium
SIRPα	signal-regulatory protein alpha
SC	segmented cells
S.C.	subcutaneous
SEM	standard error of the mean
SPF	specific-pathogen-free
SSC	side scatter
STZ	serum-treated zymosan
T:E	target:effector
TPO	thrombopoietin.
