# Supplementary material for: Humanized MISTRG as a preclinical in vivo model to study human neutrophil-mediated immune processes
Source: Front Immunol. 2023 Mar 8;14:1105103. doi: 10.3389/fimmu.2023.1105103 (PMC10032520; doi:10.3389/fimmu.2023.1105103)
Supplement: Supplementary file 1 [file DataSheet_1.docx]

Supplementary Figures


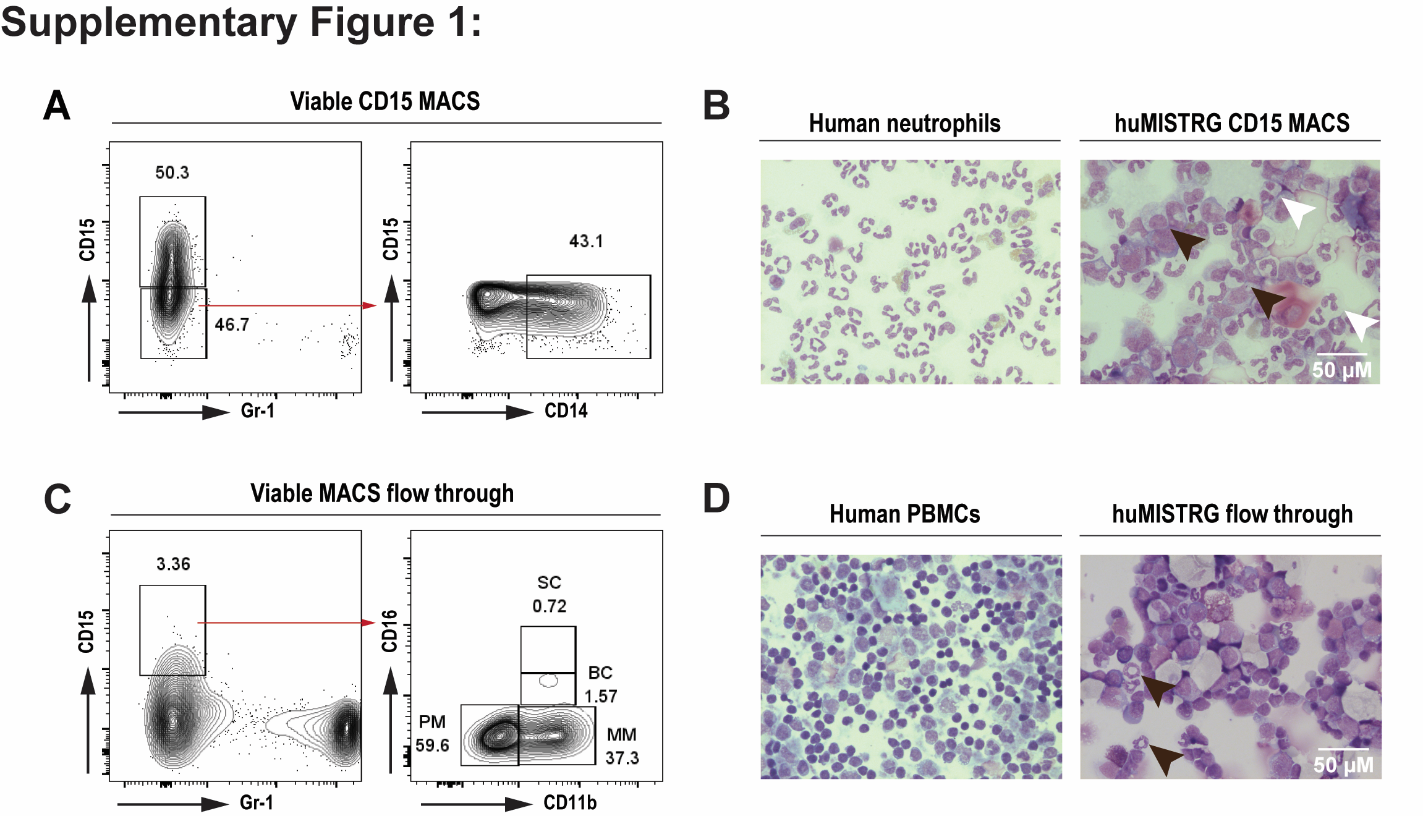


**Supplementary Figure 1.** **(A)** Gating strategy of huMISTRG samples showing presence of CD14^+^ cells within the CD15^low^ MACS-sorted fraction. Numbers indicate the percentages of the different populations. **(B)** Representative cytospin of human neutrophils (left) and CD15 MACS-sorted fractions of huMISTRG samples (right) after May-Giemsa staining (objective 50x). Black arrows indicate immature neutrophil subpopulations with round/banded nucleus, white arrows indicate mature subpopulations with segmented nucleus in huMISTRG samples. **(C)** Gating strategy of huMISTRG samples showing absence of CD15^+^ cells as well as absence of neutrophil BM subpopulations based on CD11b and CD16 staining (PM, MM, BC, SC) in the discarded flow through after MACS sorting. Numbers indicate the percentages of the different populations.  **(D)** Representative cytospin of human PBMCs (left) and flow through fraction after MACS sorting of huMISTRG samples (right) after May-Giemsa staining (objective 50x). Black arrows indicate murine neutrophils with circular nucleus in huMISTRG BM samples. PB, peripheral blood; BM, bone marrow; PBMC, peripheral blood mononuclear cells.


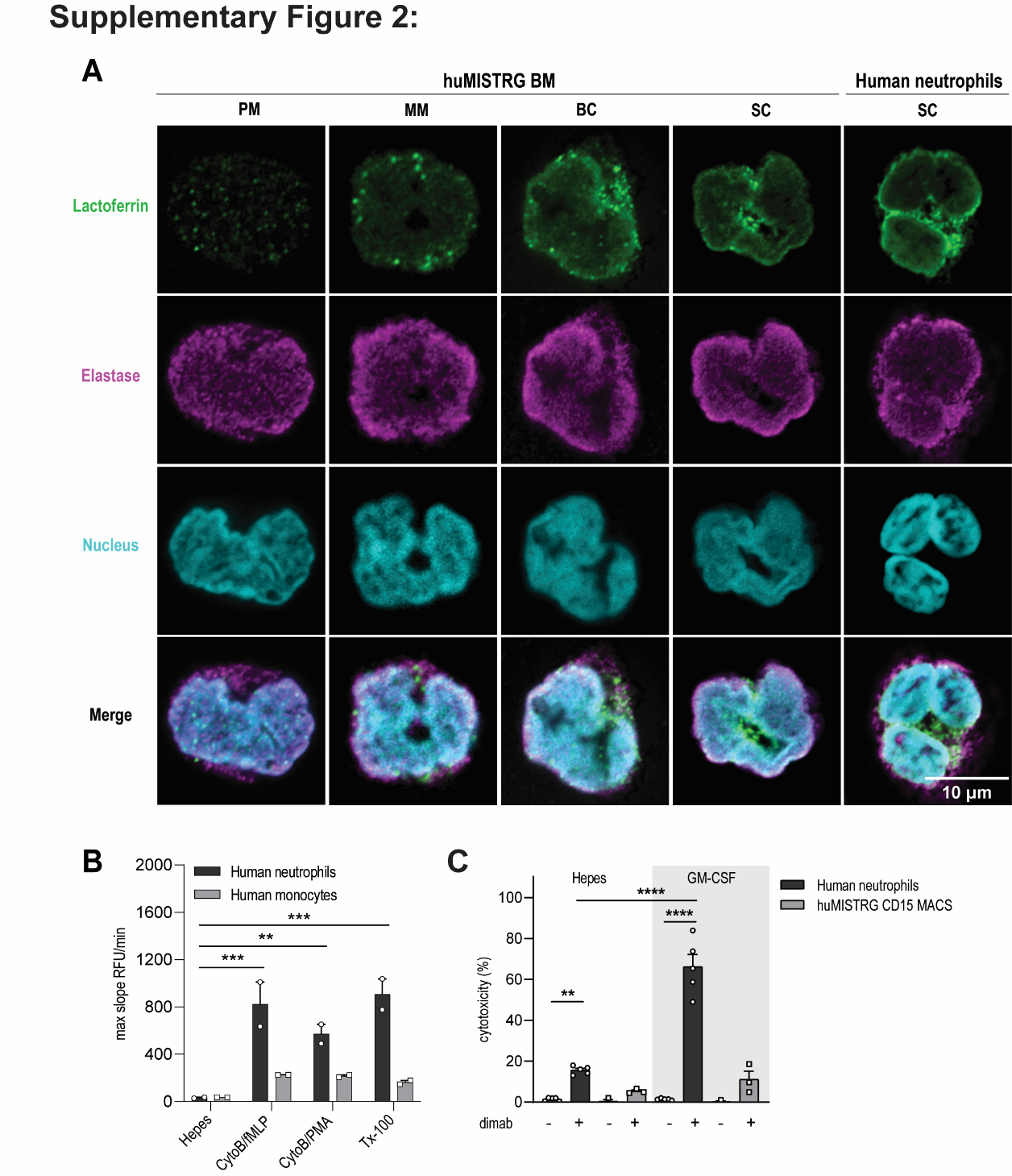


**Supplementary Figure 2. (A)** Representative confocal fluorescent image of each flow cytometry sorted neutrophil bone marrow progenitor (PM, MM, BC, SC) stained for specific granule marker lactoferrin (green), azurophilic granule marker neutrophil elastase (magenta), and nuclear marker (Hoechst, cyan). Human control samples of circulating neutrophils were used as control. **(B)** Protease activity of human neutrophils (black bars) and human monocytes (isolated by MACS sorting for CD14, grey bars) from human PB in the presence of the indicated stimuli, or Triton (Tx-100) for total release, expressed as max slope RFU/min. N=2, of one individual experiment. **(C)** ADCC of NMB neuroblastoma cells opsonized with (+) or without (−) dinutuximab (dimab) by unstimulated (Hepes) or GM-CSF stimulated human neutrophils (black bars) and CD15 MACS-sorted huMISTRG neutrophils (grey bars). N=3-5, of three individual experiments. ADCC, antibody-dependent cellular cytotoxicity; PB, peripheral blood; BM, bone marrow; RFU, relative fluorescent units.


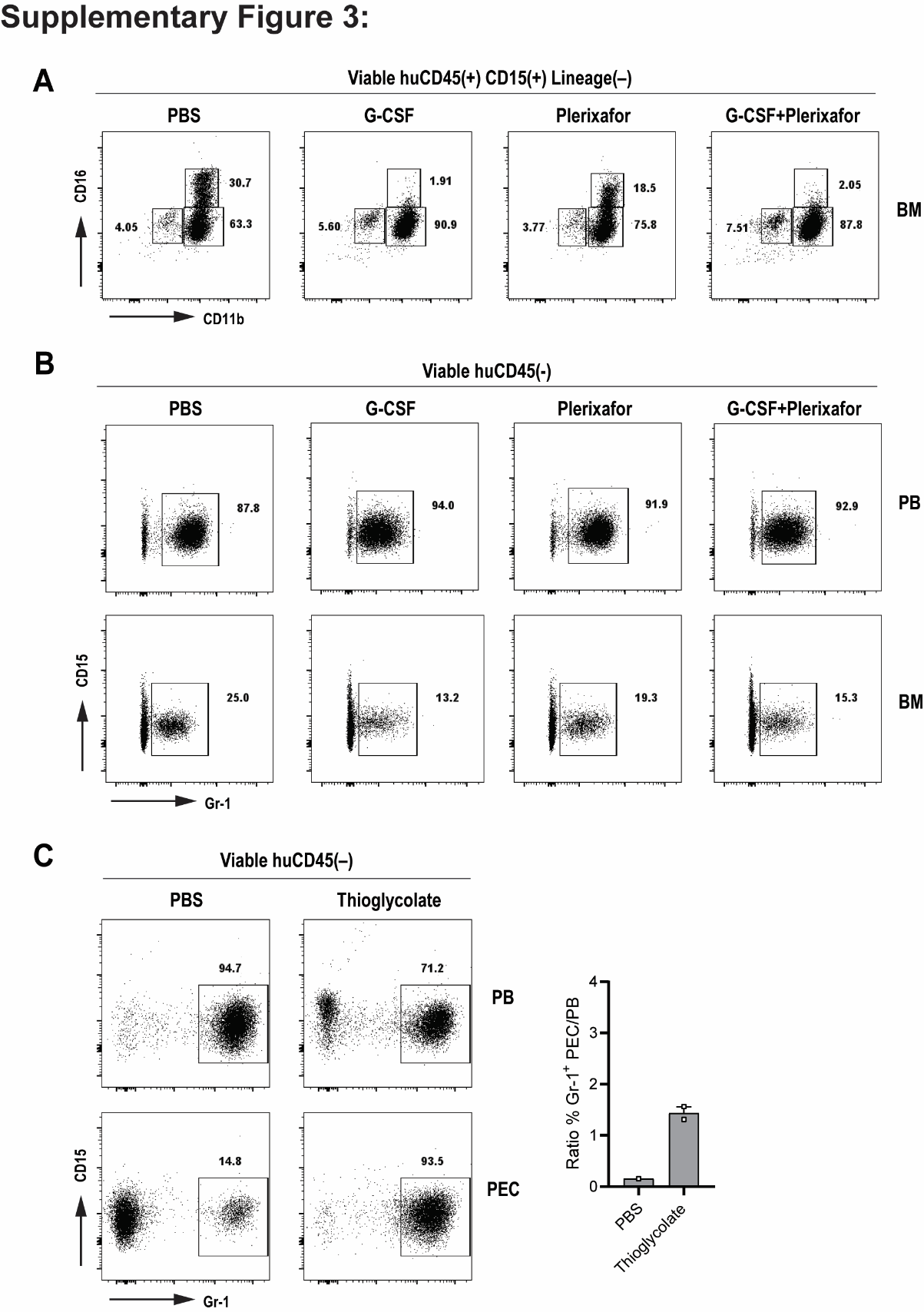


**Supplementary Figure 3. (A)** Representative CD11b vs CD16 flow cytometry plots of BM samples in response to different mobilizing agents, gated on viable human CD45^+^CD15^+^ cells. Numbers indicate the percentages of the different subpopulations. **(B)** Representative Gr-1 vs CD15 flow cytometry plots of PM (top panels) and BM (bottom panels) samples in response to different mobilizing agents, gated on viable human CD45^–^ cells. Numbers indicate the percentages of the different subpopulations. **(C)** Representative CD15 vs Gr-1 flow cytometry plots of G-CSF mobilized mice in response to peritoneal injection of thioglycolate, gated on viable human CD45^–^ cells. Numbers indicate the percentages of the different subpopulations. On the right, quantification of the influx of murine neutrophils in the peritoneum per condition represented by the ratio of Gr-1^+^ cells in the PEC suspension to those in the PB. N=1-2, of two individual experiments. BM, bone marrow; PB, peripheral blood; PEC, peritoneal exudate cells.


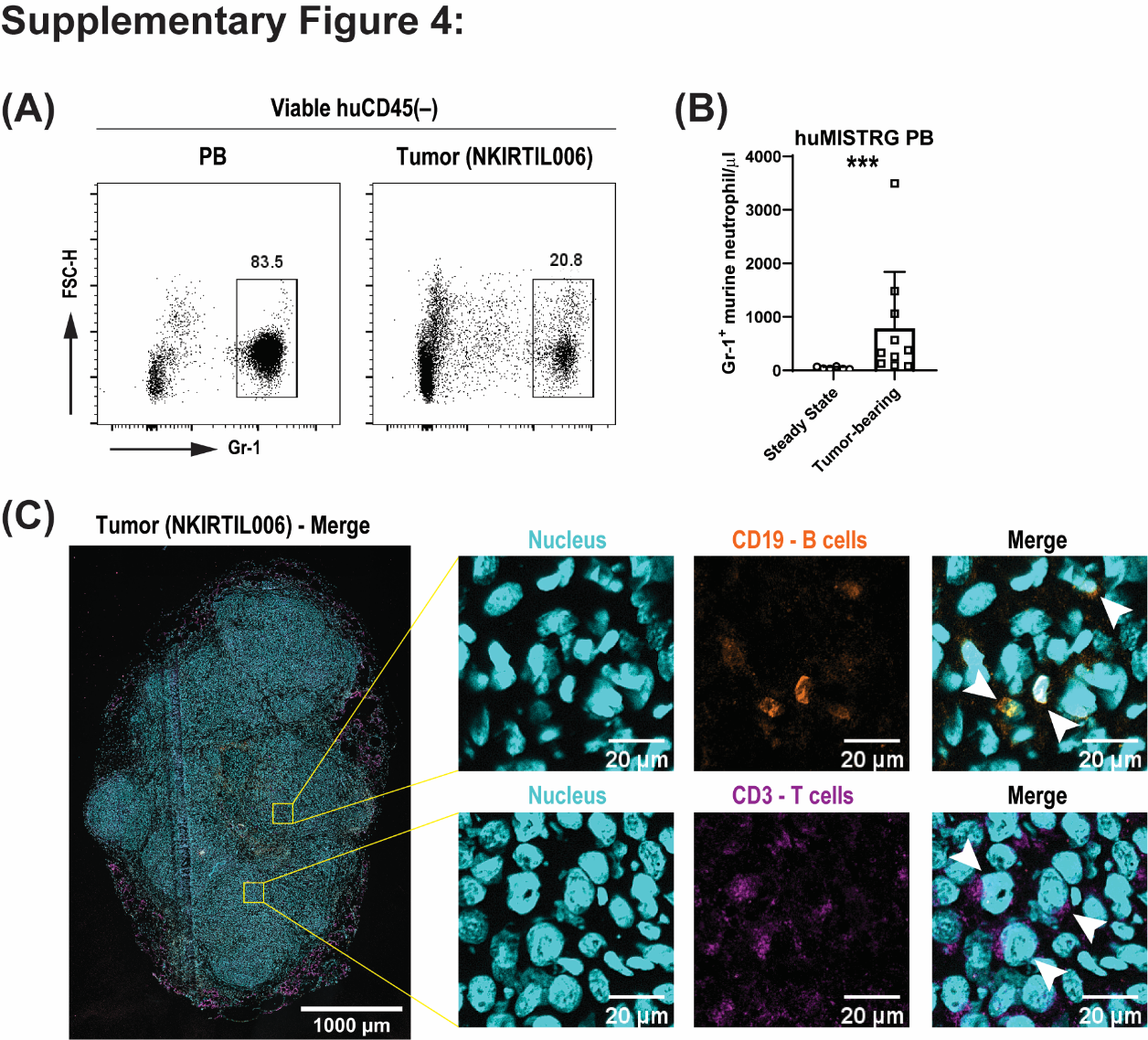


**Supplementary Figure 4. (A)** Representative Gr-1 vs FSC-H flow cytometry plots of PB or NKIRTIL006 tumor tissue samples of tumor-bearing mice, gated on viable human CD45^–^ cells. Numbers indicate the percentages of the different subpopulations. **(B)** Quantification of blood-circulating Gr-1^+^ murine neutrophils at steady state, and in tumor-bearing huMISTRG animals. N=6-10, of 2 independent experiments. **(C)** Representative wide-field fluorescent image of a 10 µm NKIRTIL006 tumor section stained for human B cells (CD19, orange), T cells (CD3, magenta) and nuclear marker (Hoechst, cyan). Crops (right) were taken from the indicated Tilescan areas of the entire tumor. White arrows indicate respective CD19 and CD3 positive staining surrounding a round nucleus, characteristic nuclear morphology of human lymphocytes.
